# An Instrumented Hammer to Detect the Rupture of the Pterygoid Plates

**DOI:** 10.1007/s10439-024-03596-9

**Published:** 2024-08-22

**Authors:** Manon Bas dit Nugues, Leo Lamassoure, Giuseppe Rosi, Charles Henri Flouzat-Lachaniette, Roman Hossein Khonsari, Guillaume Haiat

**Affiliations:** 1 CNRS, Univ Paris Est Creteil, Univ Gustave Eiffel, UMR 8208, MSME, 61, Avenue du Général de Gaulle, 94010 Créteil Cedex, France; 2 APHP, Hôpital Henri-Mondor, Service de Chirurgie Orthopédique, 94010 Créteil, France; 3 Univ Paris Est Creteil, Univ Gustave Eiffel, CNRS, UMR 8208, MSME, 94010 Créteil, France; 4 INSERM U955, IMRB Université Paris-Est, 51 avenue du Maréchal de Lattre de Tassigny, 94000 Créteil, France; 5 Service de Chirurgie Orthopédique et Traumatologique, Hôpital Henri Mondor AP-HP, Université Paris-Est, CHU Paris 12, 51 avenue du Maréchal de Lattre de Tassigny, 94000 Créteil, France; 6 APHP, Hôpital Necker-Enfants Malades, Service de Chirurgie maxillo-faciale et chirurgie plastique, Laboratoire ‘Forme et Croissance du Crâne’, 75015 Paris, France

**Keywords:** Osteotomy, Impact hammer, Bone biomechanics, Biomechanical testing

## Abstract

**Purpose:**

Craniofacial osteotomies involving pterygomaxillary disjunction are common procedures in maxillofacial surgery. Surgeons still rely on their proprioception to determine when to stop impacting the osteotome, which is important to avoid complications such as dental damage and bleeding. Our group has developed a technique consisting in using an instrumented hammer that can provide information on the mechanical properties of the tissue located around the osteotome tip. The aim of this study is to determine whether a mallet instrumented with a force sensor can be used to predict the crossing of the osteotome through the pterygoid plates.

**Methods:**

31 osteotomies were carried out in 16 lamb skulls. For each impact, the force signal obtained was analysed using a dedicated signal processing technique. A prediction algorithm based on an SVM classifier and a cost matrix was applied to the database.

**Results:**

We showed that the device could always detect the crossing of the osteotome, sometimes before its occurrence. The prediction accuracy of the device was 94.7%. The method seemed to be sensitive to the thickness of the plate and to crack apparition and propagation.

**Conclusion:**

These results pave the way for the development of a per-operative decision support system in maxillofacial surgery.

## Introduction

Osteotomes are surgical tools with a sharp blade aiming at cutting bone and cartilaginous tissues. They are impacted by a surgical mallet to perform various osteotomies such as rhinoplasty [1, 2] or sinus lift elevation [3, 4]. In particular, Le Fort osteotomies in maxillofacial surgery consist in mobilizing the maxillary and/or zygomatic bones at various levels in order to reposition the midface and correct facial deformations [5–7]. In Le Fort procedures, as well as in surgical maxillary expansion [8], the surgeon must detach the palatine bones from the pterygoid plates that are part of the skull base. In fact, these plates correspond to the pterygoid processes of the sphenoid bone. The pterygoid plates are divided into medial and lateral plates forming a V-shape [9]. At the extremity of each medial plate, a hook named ‘hamulus’ points towards the oral cavity. The pterygoid plates are attached to the palatine bone and connect the midface with the skull base. In order to cut these plates, the surgeon must slide the osteotome along the maxillary bone. Like many other osteotomies, Le Fort osteotomies are performed without direct visual control. Surgeons usually insert a finger in the patient mouth posterior and medial to the maxillary tuberosity to feel the hamulus through the mucosa. Then, the osteotome is impacted with a hammer until the tip of the osteotome can be felt by the finger placed in the mouth [5, 8, 9].

The surgeon must, therefore, rely on his/her proprioception and in particular on the sound produced by the impacts of the hammer on the osteotome in order to decide whether to stop or to continue impactions. This subjective decision requires experience. A compromise must be found between strong impacts, which could result in excessive displacement of the osteotome and lead to haemorrhage, nerve damage, and mucosal perforation [10, 11], and weak impacts that do not allow to cut bone tissue.

Different solutions have been proposed to guide the surgeon during this surgery. One solution consists in tracking the position of the osteotome with the help of 3D localization procedures coupled with X-ray-based imaging methods [12–14]. However, such approaches increase the operating time and cost and are sources of radiations for the patient and the surgeon. Alternatives to osteotomes, such as oscillating saws [15] or piezotomes [16–18] (piezo-electric cutting system) are not ideal without visual control and are mostly used for the other steps of Le Fort osteotomies [19].

In this context, our group has developed a new measurement technique that allows to carry out controlled osteotomies without modifying the surgical protocol. Like navigation systems, our approach aims at providing assistance to surgeon performing an osteotomy. However, while navigation systems allow to determine the anatomical location of the osteotome, the present approach allows to assess the two following complementary information: (i) the bone rigidity around the osteotome tip and (ii) whether a fracture occurs in the bone located around the osteotome tip. First, while navigation system may provide information on the anatomy, they cannot be used to determine bone rigidity. Bone rigidity is a mechanical property difficult to be assessed using X-ray-based techniques, which provide information on bone mineralization only. Moreover, navigation system cannot be used to provide information on the occurrence of the bone fracture in real time. Therefore, both techniques are highly complementary because the instrumented hammer can give mechanical properties on the local material being osteotomized, while navigation systems can provide information on the anatomical position of the osteotome. Moreover, an advantage of our approach lies in that (i) it does not change the clinical routine; (ii) it is does not significantly extend the procedure duration which limits the risk of infection [20]; and (iii) it is probably cheaper than using a navigation system. The use of a technique based on vibration analyses to assess bone integrity is supported by the fact that it has been widely adopted to test osteointegration in the field of dental implants [21].

This method derives from previous works aiming at monitoring the insertion of the acetabular cup implant [22–26] and of the femoral stem [27–30] during total hip arthroplasty. The approach is based on an instrumented hammer equipped with a force sensor located on its impacting face, allowing to record the signal corresponding to the time variation of the force between the hammer and the osteotome during each impact. This signal has been shown to provide information on the mechanical properties of the impacted system. A first study [31] on osteotomy has shown that the instrumented hammer can be used to: (i) determine the material of the sample and (ii) estimate the thickness of a sample with an error lower than 10%. Further studies carried out in an ex vivo animal model [32] and with anatomical subjects [33, 34] have shown that our approach can be used in rhinoplasty to detect crack initiation and bone changes around the osteotome tip. A more recent study proved that the instrumented hammer can be used to predict the rupture of plate-like bone and plywood samples when the osteotome is located at approximately 2.6 mm from the end of the sample [35]. However, the geometry of the samples used in [35] as well as its material (calf trabecular bone and plywood) were very simple and quite far from clinical reality.

The aim of the present ex vivo study is to determine the performance of an instrumented hammer to predict the rupture of a pterygoid plates being osteotomized. To do so, thirty-one osteotomies were carried out until rupture on sixteen lamb skulls using the instrumented hammer. Visual and haptic controls were performed to determine the impact corresponding to the sample rupture. A dedicated signal processing technique was developed to predict the sample rupture and the results were compared with the results obtained with visual control.

## Materials and Methods

### Experimental Material

**Fig. 1:**
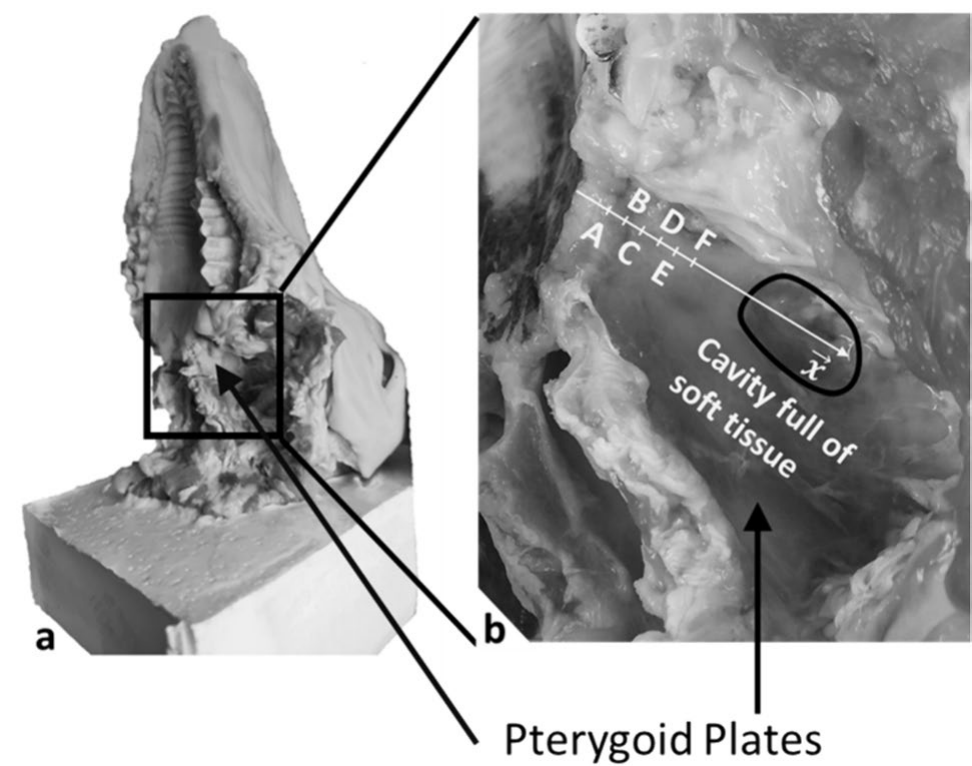
Lamb pterygoid plates: **a** Full head prepared and included in resin; **b** Zoom on the pterygoid plates, each letter, separated by 3 mm, corresponded to the location of the osteotome considered during the experimental protocol

Sixteen lamb skulls were obtained from the local butcher shop from animal slaughtered at the same age range (4 ± 1 months). Meanwhile, there is no guarantee that all skulls are similar, the dispersion being related to the biological variability. Note that we cannot control such inter-individual variability when working with biological samples. This experimental protocol was approved by and performed in accordance with the guidelines of the ethical committee of the Universite Paris-Est Creteil (UPEC). While the samples bone density may vary, all samples were prepared similarly. The 16 heads were frozen for storage and defrosted 12 hours before the experiment was started. The upper jaw was carefully removed to provide visual access to the sample during the osteotomy. All soft tissues were removed until the pterygoid plate was visible. We then checked that the pterygoid plate did not show any sign of deterioration. Then, the first vertebra was placed into a square mould and polyurethane resin (Smooth-Cast 300, Smooth-on, Pennsylvania, USA) was poured to obtain a good fixation of the sample into the vice (Fig. 1). After the resin was solidified, we carefully removed it from the mould to carry out the experiments and it was fixed firmly in a vice. The osteotome had a 6-mm curved edge (CP304B-6, Microfrance, France), similar to instruments used for Le Fort osteotomies designed to reach the pterygoid plates by getting around the maxillary bone.

**Fig. 2:**
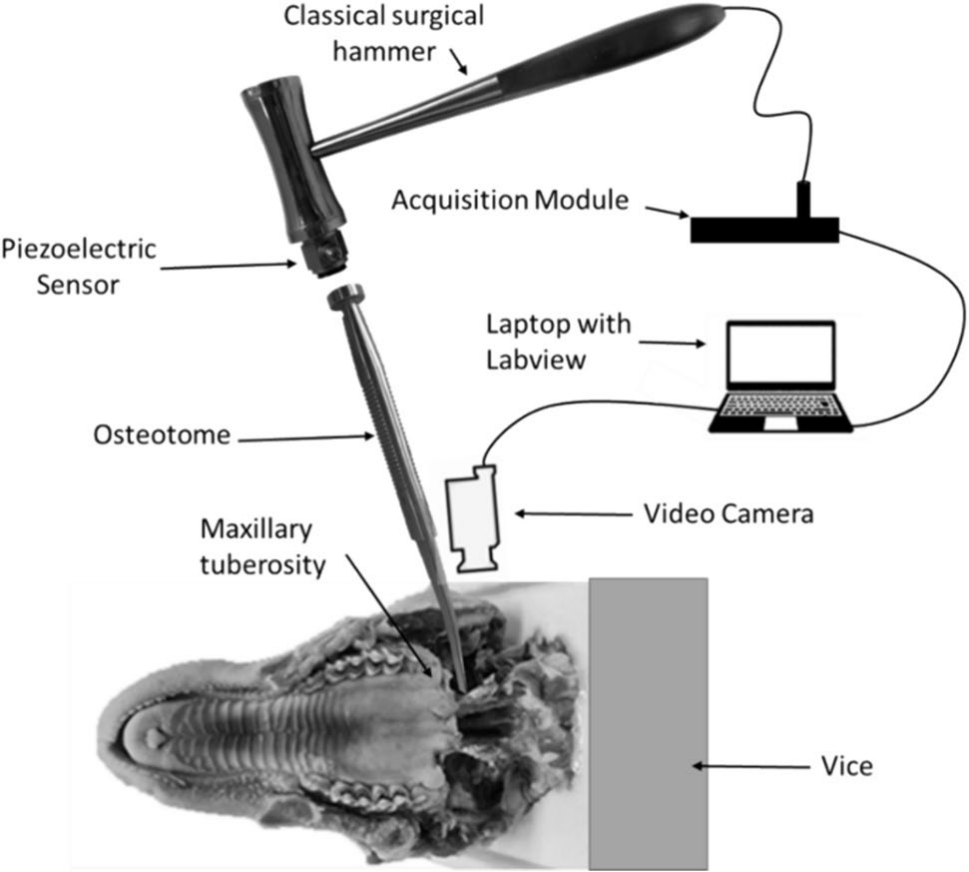
Experimental configuration considered during the osteotomy including the camera, the bone sample, the osteotome and the instrumented hammer. The impacts realized with the hammer were performed in a direction parallel to the axis of the osteotome

**Fig. 3:**
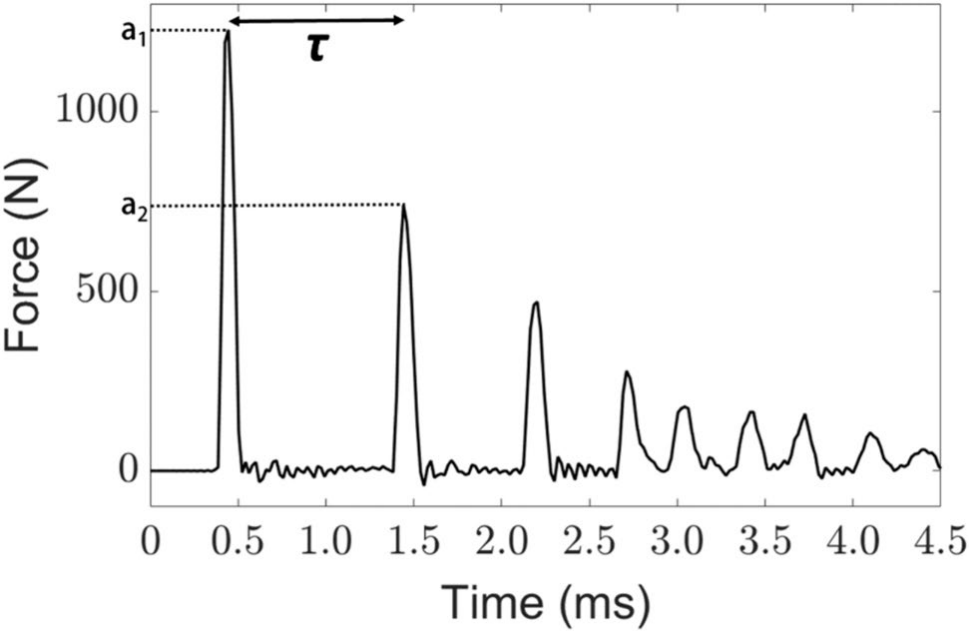
Example of a part of the signal *s(t)* corresponding to the variation of the force as a function of time obtained for a given impact of the instrumented hammer on the osteotome. *τ* denotes the time between the two first peaks of the signal. *a*_*1*_ and *a*_*2*_ correspond to the values of the maximum amplitude of the first and second peaks, respectively.

The experimental set up is shown in Figure 2. The instrumented mallet used was the same as the one described in [32, 33, 35]. It was composed of a common classical surgical mallet (32-6906-26, Zepf, Tuttlingen, Germany) weighing 260 g on which a piezo-electric force sensor (208C04, PCB Piezotronics, Depew, NY, USA) was screwed. This sensor had a measurement range up to 4.45 kN in compression and a sensitivity of 1124 mV/kN. A data acquisition module (NI 9234, National Instruments, Austin, TX, USA) with a sampling frequency of 51.2 kHz and a resolution of 24 bits was used to record the variation of the force as a function of time for a duration of 20 ms. The sampling frequency was chosen as a compromise between a value high enough to allow discretization of the signal but not too high to avoid obtaining signals that are too large in memory. We have checked that increasing the sampling frequency does not modify the results, while it increases the computation time. A part of the signal *s(t)* corresponding to a given impact of the hammer on the osteotome is shown in Fig. 3. Similarly, as in previous studies from our group [31–33, 35], the time of the maximum of the first two peaks of *s(t)* was determined for each impact.

The indicator* τ* was then defined as the difference between the times of the second and first peaks of *s(t)*. Each peak *#p* (*p* = 1, 2) was approximated by a Gaussian fit and the values of the maximum peak amplitude *a*_*p*_ and of the Gaussian root-mean-square width *w*_*p*_ were calculated. *w*_*p*_ is proportional to the width at − 6 dB of the Gaussian function. *L*_*p*_ was defined as *L*_*p*_* = (2π)*^*0,5*^*. w*_*p*_* . a*_*p*,_ following what has been done in [31]. The impulse ratio *λ* was then determined by the ratio between the first two peak integrals *L*_*1*_ and *L*_*2*_, i.e. *λ = L*_*2*_*/ L*_*1*_.

A video camera (L-920M3, Spedal, Taiwan) was used to monitor crack apparition. To be as close as possible of the clinical situation, the hammer and the osteotome were held manually.

### Experimental Procedure

Two steps could be distinguished in the experimental protocol. Firstly, we performed a ‘thickness assessment’ procedure in order to determine whether the value of *τ* could be used to assess the sample thickness around the osteotome location. Then, we performed an ‘osteotomy’ procedure in order to cut the bone and determine whether the instrumented hammer could be used to assess the sample rupture.

The x-axis was determined based on the anatomy of the head and on the surgeon proprioception by following the maxillary tuberosity (see Fig. 2). The osteotomy and the thickness assessment procedures were then both performed by successively moving the osteotome along the x-axis similarly as in clinical practice where the osteotomy should be performed as close as possible to the junction between the palatine bone and the pterygoid plates*.*

It should be noted that the first position of the osteotome (#A in Fig. 1b) was not completely at the end of the plate because of the presence of soft tissues.

During the thickness assessment procedure, the osteotome was first positioned between the first position (#A in Fig. 1b) and the third position (#C in Fig. 1b). Five impacts with a relatively low energy, (which correspond to a maximum amplitude of *s(t)* comprised between 100 and 200 N) were applied with the hammer on the osteotome. These impacts with a relatively low energy allowed to measure the impact response without risking to damage the plate nor to create any crack. The osteotome was then translated along the x-axis by a distance corresponding to half the length of the blade (i.e. 3 mm) and five similar impacts were reproduced. The similar procedure (i.e. translation and realization of five impacts) was reproduced until the osteotome reached the cavity full of soft tissue (see Fig. 1b). The average and standard deviation values of *τ* were determined for each osteotome position. Each position of the osteotome during the thickness assessment procedure will be referred to as ‘thickness assessment location’ in the following.

During the osteotomy procedure, the osteotome was first positioned between the first position (#A in Fig. 1b) and the third position (#C in Fig. 1b). The osteotome was impacted with the instrumented hammer until it crossed the plate, which was determined by the operator who placed a finger under the plate, similarly as what is done in the operating room. An additional impact was given to make sure that the osteotome was in contact with air at the end of the procedure (since soft tissue had been removed). The osteotome was then translated along the x-axis by a distance corresponding to the length of the blade (i.e. 6 mm). If no crack was present in front of the osteotome, it was again impacted until the plate failure. If not, the osteotome was again translated along the x-axis until no crack was present and it was again impacted until the plate failure. Therefore, the osteotomy location did not necessarily correspond to the location of the thickness assessment. The same procedure was applied until the osteotome reached the cavity full of soft tissue (see Fig. 1b). Each position of the osteotome during the osteotomy procedure will be referred to as ‘osteotomy location’ in the following. The total number of impacts realized during the osteotomy procedure for each plate will be noted *N*.

### Thickness Measurements

After the osteotomy procedure was performed, each pterygoid plate was entirely removed from the head by using the same osteotome and a non-instrumented mallet (32-6906-26, Zepf, Tuttlingen, Germany). The variation of its thickness along the x-axis was assessed using a digital calliper (AEI-CAL-2, AEI-ITC, Lillois, Belgium).

The plate thickness was assessed 5 times every 2.5 mm along the x-axis to (i) evaluate the average thickness of the plate at each position corresponding to each measure during the thickness assessment and the osteotomy and (ii) assess the reproducibility of the measurement realized with the calliper. These data were then gathered to correspond to each measure made during the thickness assessment and the osteotomy. For example, all the measurements between positions #A and #C were averaged to obtain the thickness of the plate for the first thickness assessment location.

### Regression Analyses

For the thickness assessment and osteotomy procedures, the variation of the indicator *τ* as a function of the thickness of the plate could be divided into two regimes. First, *τ* remained constant when the thickness was high enough. Then, when the thickness decreased, *τ* seemed to increase. As it has already been reported in [35], we assumed a linear by part dependence of *τ* as a function of the thickness *Th* following:

1 $$\widetilde{\tau }\left(Th\right)= \left\{\begin{array}{c}{ \tau }_{0} if Th>{d}_{f}\\ {\tau }_{0} + {k}_{f}.\left(Th-{d}_{f}\right) if Th \le {d}_{f}\end{array}\right.,$$

where *d*_*f*_ was the value of the thickness delimiting the two different regimes, *k*_*f*_ was the slope of the curve representing the variation of *τ* as a function of the thickness during the second regime, and *τ*_*0*_ was the value of *τ* during the plateau (first regime).

Based on the experimental results, a cost function *e*_*τ*_* (d*_*f*_*, k*_*f*_*, τ*_*0*_*)* was defined to assess the difference between the experimental measurements and values given by Eq. 1 following:

2 $${e}_{\tau } \left({d}_{f},{k}_{f},{\tau }_{0}\right)= \sum_{j=1}^{P}\frac{\left|\tau \left(j\right)-\widetilde{\tau }\left(Th(j\right))\right|}{P},$$

where* j* corresponded to the number of the location of the thickness assessment (respectively osteotomy) procedure, *P* to the total number of locations made for all the thickness assessments (respectively osteotomies), *τ(j)* corresponded to the value of *τ* for the location *#j* and *Th(j)* corresponded to the measured thickness of the plate for the location *#j*.

An optimization procedure based on the Nelder–Mead method [36] was carried out to determine the optimal values of the parameters *(d*_*f*_*, k*_*f*_*, τ*_*0*_*)* minimizing the cost function *e*_*τ*_ for each sample. The minimum value of *e*_*τ*_ obtained by the optimization procedure was noted *E*_*τ*_.

This linear regression by part (see Eq. 1) and optimization procedure (see Eq. 2) were applied to determine the values of the parameters *d*_*f*_, which corresponded to the minimum plate thickness leading to an increase of *τ*.

### Data Processing

#### Creation of the Database

Once the data were gathered and processed as indicated in the previous subsections, a database was created based on all impacts realized for all osteotomies. For each impact *#i*, *1 < i ≤ N*, the values of the indicators *τ(i)* and *λ(i)* were determined, as well as the parameters *∆τ(i) = τ(i) − τ(i − 1)* and *∆λ(i) = λ(i) − λ(i − 1)* corresponding to the variations of *τ* and *λ* compared to the previous impact. Two classes of impact were then considered, as in [33].

- The impact was considered to belong to the class referred to as ‘Bone’ if the osteotome did not cross the bone plate.
- The impact was considered to belong to the class referred to as ‘Crossing’ when the operator was able to feel the osteotome crossing the plate. This group also included the additional impacts mentioned in “Experimental Procedure” section given immediately after the rupture of the bone plate.

An ANOVA Analysis was performed to determine whether *τ* was statistically different between the classes ‘Bone’ and ‘Crossing’. The canonical variables *c*_*1*_ and *c*_*2*_ were also determined following [37] and corresponded to linear combinations of the indicators *τ, λ, ∆τ*, and *∆λ* allowing to maximize the distance between the data groups. The database was, thus, composed of three quantities: *c*_*1*_, *c*_*2*_, and the class of the impact.

Since the purpose of our device was to detect the impacts labelled as ‘Crossing’, we have developed a classification algorithm based on a Support Vector Machines (SVM) algorithm coupled with a cost matrix described below. The interest of the cost matrix was to minimize the number of false-negative events, which are highly detrimental for the surgeon because it corresponds to an undetected ‘Crossing’ event.

#### Support Vector Machine Algorithm

Support vector machines (SVM)-based methods are learning techniques introduced by Vladimir Vapnik in the early 1990 s as described in [38] for binary classification and regression. SVM methods are commonly used in many fields such as medicine as shown in [39, 40]. A SVM classifier looked for the hyperplane with the largest margin (i.e. distance between the boundary and the closest points of each class).

We have chosen to perform a soft margin SVM classifier with a linear kernel as our data were not linearly separable [41]. To avoid overfitting, we performed a k-fold cross validation (with *k* = 5). This means that for each of the five loop, the algorithm was trained with four folds randomly selected from the database as training data then the classifier was validated on the remaining unknown part of the database. The accuracy performance was provided by the average of the value measured on each loop. Since we aimed at developing a tool warning the surgeon when the osteotome crossed the plate, the negative event was defined as an impact classified as ‘Bone’ and the positive event as an impact classified as ‘Crossing’. Therefore, a False Negative, (respectively False Positive) event corresponded to an impact belonging to the class ‘Crossing’ (respectively ‘Bone’) and classified as ‘Bone’ (respectively ‘Crossing’) by the SVM method. In the following, FN, FP, TN, and TP will correspond to False Negative, False Positive, True Negative, and True Positive, respectively.

#### Optimization of the Classification Algorithm

Since the purpose of our device was to detect the impacts labelled as ‘Crossing’, a cost matrix was added into the SVM classifier described above, similarly to what was done in [42]. The aim of this cost matrix was to reduce the number of FN, even if it implied a small increase of the number of FP. The cost matrix defined in both studies [42, 43] writes

3 $$\sigma = \left[\begin{array}{cc}{\sigma }_{BB}& {\sigma }_{CB}\\ {\sigma }_{BC}& {\sigma }_{CC}\end{array}\right]$$

where the subscript *C* indicates ‘Crossing’ and *B* indicated ‘Bone’. Each coefficient *σ*_*lm*_ represents the relative weight applied for the misclassification of an impact actually belonging to the class ‘*l*’ and classified by the prediction algorithm in the class ‘*m*’. The aim here was to penalize FN more than FP in order to reduce their numbers.

The values of the coefficients *σ*_*CC*_, *σ*_*CB*_, *σ*_*BC*_, and *σ*_*BB*_ were determined using an optimization method detailed in [44]. This optimization method was based on the Negative Likelihood Ratio (NLR). This ratio was defined as in Eq. 4 and in the study [45] to assess the performance of a diagnostic test:

4 $${\text{NLR}}= \frac{{\text{FN}}/({\text{TP}}+{\text{FN}})}{{\text{TN}}/({\text{FP}}+{\text{TN}})}.$$

During the optimization of the coefficients, the algorithm tried to minimize this metric. This will, therefore, lead to a decrease of the number of FN while preserving a maximum of TN.

All the data were gathered and processed with Matlab (MATLAB, R2017b, MathWorks, Natick, Massachusetts, USA).

## Results

### Number of Impacts

The osteotomy procedure results in 3.52 ± 0.72 locations with a total number of impacts *N* = 54.97 ± 18.91. Table 1 describes in more details the statistics on the number of osteotomy locations and impacts obtained for all 31 samples. A decrease of the number of impacts is obtained as a function of the osteotomy location number, which may be explained by a weakening of the structure. Regarding the thickness assessment procedure, 5.65 ± 0.98 locations are obtained for each sample.

**Table 1:** Statistics of the number of osteotomy locations and of the number of impacts per location

Osteotomy location #	Number of occurrences (total: 31)	Average number of impacts	Standard deviation
1	31	14.60	± 12.82
2	31	10.32	± 4.66
3	29	7.83	± 3.60
4	16	7.94	± 3.32
5	2	8.00	± 2.83

### Results on the Sample #12G

**Fig. 4:**
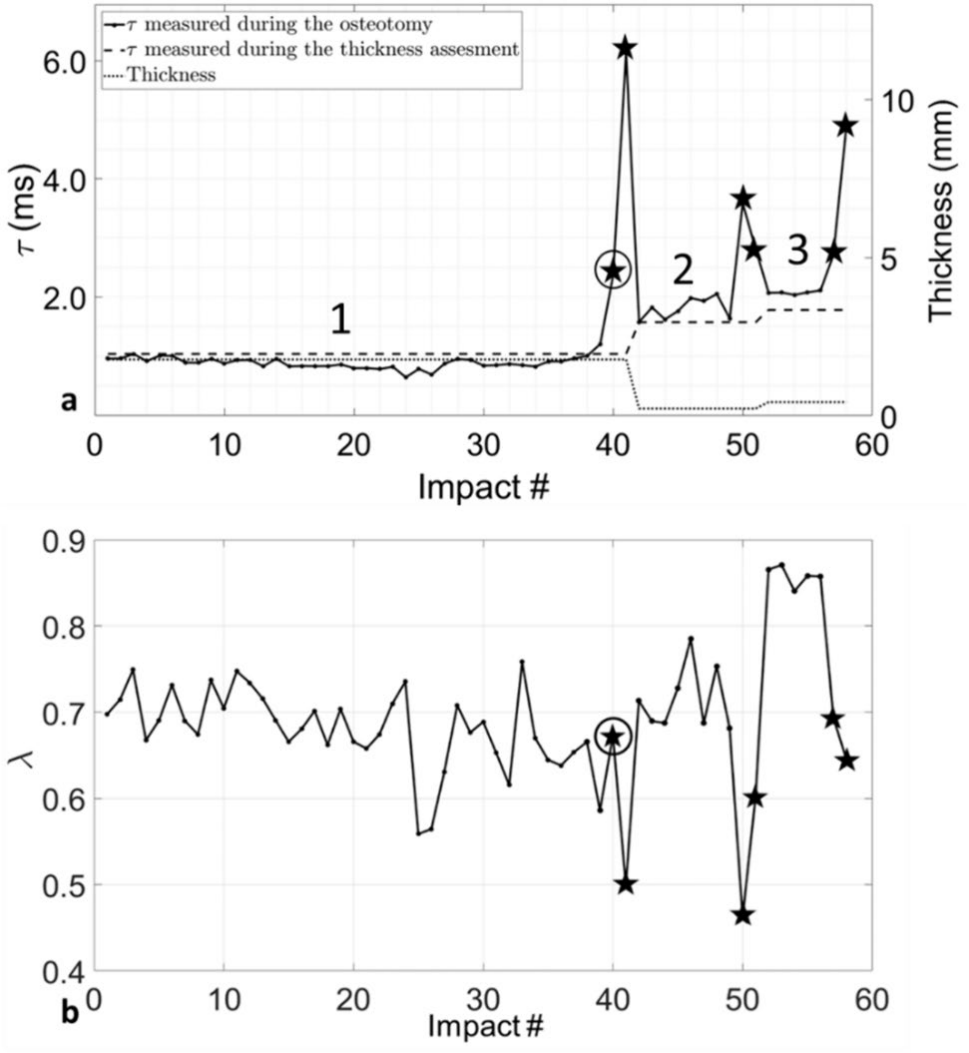
Result obtained for the sample #12G: **a** Solid line: variation of *τ* as a function of the impact number for the three locations of the osteotome indicated by the numbers. Dashed line: Average value of *τ* obtained during the thickness assessment corresponding to the location of the osteotome. Dotted line: the thickness of the plate corresponding to the location of the osteotome. **b** Variation of *λ* along the osteotomy of the sample. The stars correspond to the impact classified as ‘Crossing’ by the classification algorithm, and the prediction errors are indicated with a circle

Figure 4 shows the variation of *τ* and *λ* as a function of the number of impacts during an osteotomy procedure corresponding to the sample #12G. Figure 4a shows the variation of *τ* during the thickness assessment as well as the thickness measured corresponding to each position. The impacts represented by stars correspond to the impacts labelled as ‘Crossing’ by the classification algorithm.

Figure 4a shows that three osteotomy locations can be distinguished for the sample #12G. The first (respectively second and third) location corresponds to impact numbers comprised between #1 and #41 (respectively, #42 and #51, #52 and #58). The behaviour of *τ* is similar for each location since *τ* initially stays constant and then increases suddenly when fracture occurs. Moreover, the constant value of *τ* increases as a function of the location number. While the behaviour of *λ* is less predictable, a sudden decrease is obtained when fracture occurs. The classification algorithm is shown to be accurate except for impact #40, which corresponds to a FP. This error is due to a sudden increase of *τ* just one impact before failure occurs.

**Fig. 5:**
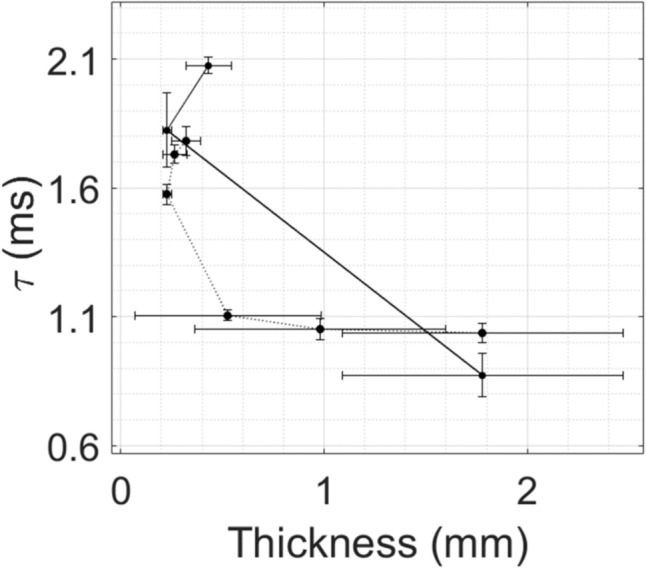
Variation of the indicator *τ* as a function of the thickness of the pterygoid plate for the osteotomy (solid line) and thickness assessment (dashed line) procedures with the sample #12G. The standard deviation of *τ* and of the thickness are also represented for each location by error bars

Figure 5 shows the variation of *τ* as a function of the thickness of the pterygoid plate measured during the osteotomy and the thickness assessment of the same sample #12G. Each point corresponds to the average value of *τ* measured along the corresponding location. This average only includes the impacts belonging to the class ‘Bone’. The standard deviation of the three first measurement of the thickness during the thickness assessment is significant, which may be explained by the fact that the thickness of the plate evolves quickly at the edge of the plate. The standard deviation of the values of *τ* obtained during the second location of the osteotomy is also more scattered, which may be explained by the presence of a crack.

Table 2 below summarizes the minimum, maximum, and average *τ* for each thickness assessment location.

**Table 2:** Average, maximum, and minimum value of *τ* for each thickness assessment location

Thickness assessment location #	Average value of *τ* (ms)	Minimum value of *τ* (ms)	Maximum value of *τ* (ms)
1	1.0372	1.0015	1.0943
2	1.0515	0.9999	1.0963
3	1.1060	1.0861	1.1339
4	1.5747	1.5281	1.6217
5	1.7311	1.6927	1.7728
6	1.7819	1.6909	1.8279

For the last osteotomy location, *τ* continues to increase while the plate thickness also increases, which may be explained by the fact that the plate is partially separated from the rest of the head, leading to a decrease of the rigidity of the plate and, therefore, to an increase of *τ* although the plate thickness slightly increased.

### Results for the Entire Database

**Fig. 6:**
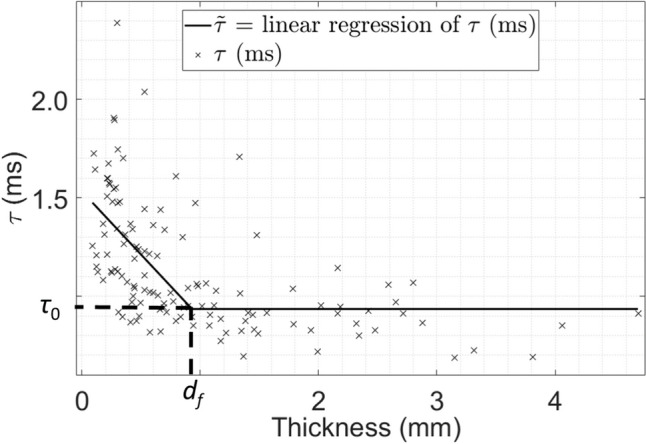
Variation of *τ* as a function of the thickness for all thickness assessment locations. The linear by part interpolation $\widetilde{\tau }$ is shown with the solid line. *d*_*f*_ corresponds to the average value of the thickness below which the plate thickness influences the value of *τ*

**Fig. 7:**
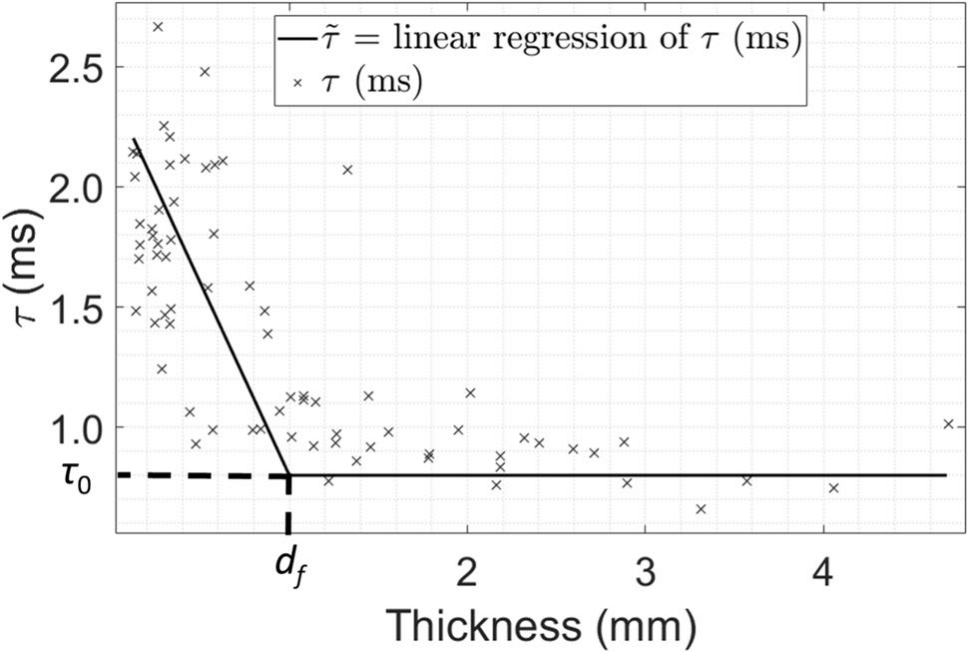
Variation of *τ* as a function of the thickness for all osteotomy locations. The linear by part interpolation $\widetilde{\tau }$ is shown with the solid line. *d*_*f*_ corresponds to the average value of the thickness below which the plate thickess influences the value of *τ*

Figures 6 and 7 show the variation of *τ* as a function of the thickness for all thickness assessment locations and all osteotomy locations, respectively. Figures 6 and 7 correspond to a full visualization of the complete database depicted by one sample in Fig. 5. Each cross in Fig. 6 (respectively Fig. 7) corresponds to the average value of *τ* for the corresponding thickness assessment location (respectively, osteotomy location) and for the impacts belonging to the class ‘Bone’. The results obtained with the linear by part regression procedure described in “Regression Analyses” subsection are also shown. Figures 6 and 7 show that* τ* is quite stable around *τ*_*0*_ until the thickness is lower than *d*_*f*_. For *τ < d*_*f*_, *τ* then increases when the thickness decreases.

Table 3 shows the values of the parameters corresponding to the two linear by part regression analyses obtained for the thickness assessment and the osteotomy procedures. Table 3 shows that the value of *E*_*τ*_ are higher for the osteotomy than for the thickness assessment, which indicates that the data are more scattered. This result may be explained by the fact that the rigidity of the plate during the osteotomy procedure is not only determined by the plate thickness but also by the presence of cracks, which appear during the procedure.

**Table 3:** Values of the parameters *τ*_*0*_, *d*_*f*_, *k*_*f*_, and *E*_*τ*_ obtained for the linear by part regression analysis applied to the thickness assessment and osteotomy locations shown in Figs. 5 and 6

Parameters	*τ*_0_ (*m*s)	*d*_*f*_ (mm)	*k*_*f*_ (ms/mm)	*Eτ*
Thickness assessment	0.9351	0.93	− 0.6423	22.4
Osteotomy	0.9507	1.2	− 0.8924	38.5

### Classification Results

The ANOVA analysis performed on the data corresponding to the osteotomy showed that the value of* τ* (respectively *λ*) is significantly lower (respectively higher) for the data labelled as ‘Bone’ compared to those labelled as ‘Crossing’ with *p* < 0.001, *F* = 125.3 (respectively *p* < 0.001, *F* = 294.3).

**Fig. 8:**
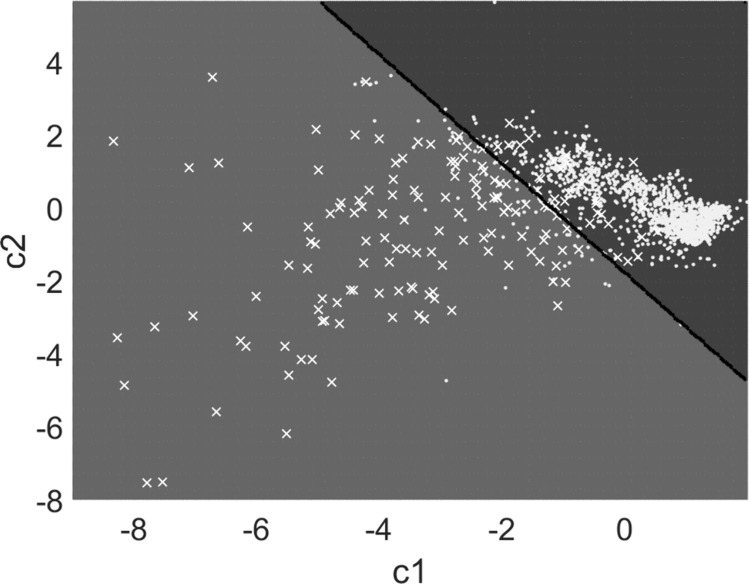
Result of the classification algorithm in the canonical variable space. Dots and crosses represent the impacts classified as ‘Bone’ and ‘Crossing’, respectively. The two different spaces represent the result of the algorithm. The light grey (respectively dark grey) space corresponds to the ‘Crossing’ (respectively ‘Bone’) space prediction. The black line corresponds to the boundary between the two spaces.

Figure 8 shows the result of the classification algorithm in the space corresponding to the canonical variables *c*_*1*_ and *c*_*2*_. The two spaces in light and dark grey correspond to the results of the classification algorithm, namely the ‘Crossing’ and the ‘Bone’ space, respectively. Dots and crosses correspond to impacts belonging to the ‘Bone’ and ‘Crossing’ classes, respectively. The black line corresponds to the limits between the two spaces.

Table 4 shows the performances of the classification algorithm. The cost matrix used here reads

**Table 4:** Statistic on the number of correct predictions (true positive and true negative), false positive, and false negative of the classification algorithm

Parameters	Result of the classification algorithm
Total impact number	1591
Correct predictions (TP + TN)	1507
False positive (FP)	55
False negative (FN)	29

5 $$\sigma = \left[\begin{array}{cc}0& 3.5\\ 1& 0\end{array}\right].$$

The accuracy of the classification algorithm is equal to 0.947. Please note that the same analysis without the indicators Δ*τ* and Δ*λ* has been performed (data not shown) and the results showed that the performances of the classification algorithm were slightly better with Δ*τ* and Δ*λ* than without.

## Discussion

The main originality of this study was to determine if an instrumented hammer used in combination with an SVM-based approach with a cost matrix could be used to indicate when the osteotome crossed the pterygoid plate, which may be useful to help maxillofacial surgeons for pterygomaxillary disjunction for various procedures such as Le Fort osteotomies.

### Indicators Obtained with the Instrumented Hammer

The simple analytical model developed in [30] was able to predict signals corresponding to the variation of the force as a function of time during an impact that were qualitatively similar to the ones shown in Fig. 3. The first peak was related to the initial impact of the hammer on the osteotome, while the following ones corresponded to the rebound of the osteotome between the bone and the hammer. Therefore, the value of *τ* was determined by the time necessary for the osteotome to reach the sample, to bounce on it and to come back to impact the hammer again. Therefore, *τ* was related to the rigidity of the material around the osteotome tip. A decrease of the rigidity of the sample, which may for instance be due to a crack propagating in front of the osteotome and/or to a decrease of the plate thickness, would then lead to an increase of *τ* [31, 33]. This explanation is in line with the results shown in Fig. 4a, which showed a sudden increase of *τ* when the osteotome crossed the plate, which corresponded to a lower rigidity of the bone-osteotome system. The increase of *τ* corresponding to the osteotome crossing the plate was also consistent with previous results [35] obtained with trabecular bone plates, which showed an increase of *τ* before and during the rupture.

The indicator *λ* was related to the viscoelasticity of the material surrounding the tip of the osteotome [31]. Although the link between the viscoelasticity of the material and the rupture of the sample remains unclear, *λ* seemed to consistently decrease when the osteotome crossed the plate. *λ* can, thus, provide additional information (see impact #39) but is not as reliable as *τ*. Indeed, some others unexpected variations of *λ* were obtained (see for example the impact #25 in Fig. 4b). Further investigations are required to understand the link between *λ* and the plate rupture. We verified that *λ* was not related to the plate thickness (data not shown). Moreover, the same measurement was performed by different operators and no significant effect of the operator was found for the values of *τ* and *λ*. To summarize, both indicators do have a physical explanation and we used a SVM classification methods with a cost matrix to distinguish the impacts based on the material on which the tip of the osteotome is located.

### Relationship Between the Indicator *τ* and the Thickness

As indicated in “Results on the Sample #12G” subsection, *τ* increased when the osteotome crossed the plate, which was in agreement with the results obtained in [35]. Moreover, Figs. 6 and 7 show that *τ* started increasing when the plate thickness decreased for the osteotomy and the thickness assessment procedures, respectively. This result can be explained by the lower rigidity of the plate when it approaches its rupture and is also in agreement with Fig. 6 of [31] and with Figs. 4 and 5 of [35], which show the same behaviour as the thickness of the material in front of the tip of the osteotome decreases. However, the values of *τ*_*0*_ (i.e. 0.9351 ms for the thickness assessment and 0.9507 ms for the osteotomy) shown in Table 3 are lower compared to what was found in calf trabecular bone (i.e. 1.1281 ms) [35]. This could be explained by the fact that the pterygoid plate is mostly composed of cortical bone. Interestingly, the values of *k*_*f*_ obtained for the osteotomy were similar than the one obtained for calf trabecular bone plates [35].

Here, the value of *d*_*f*_ obtained for the osteotomy and for the thickness assessment were lower than the ones obtained with calf trabecular bone (i.e. 2.6 mm) [35]. This could be explained by the differences in geometry and more specifically by the fact that the samples used here are thinner than 3 mm.

### Limitations

This study has several limitations. First, we chose to work on lamb skull, which is a well-known animal model in maxillofacial surgery [46], and for craniofacial distraction osteogenesis [47–49]. Sheep has two pterygoid plates on each side of the skull base, which have a planar shape at their extremity and are separated by soft tissues located between these two plates (see Fig. 2), which is significantly different from human anatomy since human pterygoid plates (i) are made of two plates both forming a V-shape, (ii) are smaller than sheep plates, and (iii) have different biomechanical properties. Future studies should be performed with human anatomical subjects to validate the present results, which was not possible before validation in an animal model due to ethical considerations. We considered an ex vivo study because our ethical committee does not allow to perform a study with living animals before testing the technique with dead tissue when possible. Note that we did not consider the use of bone mimicking phantoms because after some trials with various sawbones samples (Sawbones solid foam sheet with density varying from [10; 20; 30; 40] PCF and thickness varying from [1; 2; 3] mm), we could not obtain a steady crack propagation because the samples suddenly broke after several impacts. The fracture behaviour of polyurethane samples is very different compared to actual bone tissue. Since we work with biological samples, an inter-individual variation of mechanical properties may be observed, which is a situation similar to what is observed in the clinic.

Second, we did not investigate the effects of soft tissues since they were removed in order to work under standardized conditions. Although the influence of soft tissues was found to be negligible in a previous study [50] made in the context of hip implant surgery, the presence of soft tissue may influence the results. This question was not investigated herein and is left to future studies. We chose to remove the soft tissues in order to facilitate (i) the detection of the moment when the osteotome crosses the bone and (ii) the visualization of the apparition of the cracks. The integrity of the nerve fibre should be checked, but this point is out of scope of the present paper since we only focus on bone tissue integrity.

Third, we choose to consider for the classification algorithm a SVM classifier coupled with a cost matrix because this cost matrix allows to minimize the FN events. While using this cost matrix leads to a slight decrease of the accuracy of the prediction algorithm from 0.958 to 0.947 and to an increase of FP events from 15 to 55, it also leads to a decrease of the FN events from 49 to 29.

Fourth, as shown in Table 4, the SVM-based method was associated with several False-Positive (FP) and False-Negative (FN) classifications.

29 FN were obtained, which corresponded to impacts belonging to the class ‘Crossing’ and classified as belonging to ‘Bone’.

- For 13/29 FN, the crossing of the osteotome was detected for the following impact.
- For 5/29 FN, the crossing of the osteotome was detected in one of the five previous impacts. These misclassifications could be explained by damaged bone tissues, which led to an increase of *τ,* but not to the crossing of the osteotome.
- However, for 11/29 FN, the crossing of the osteotome was not detected. For 9 of these 11 cases, the value of *τ* did not increase enough to detect the crossing of the osteotome, which could have been the case if additional impacts had been given. However, we chose to only apply one impact after the osteotome crossed the plate to avoid excessive damage to the structure. The 3 other FN out of these 11 cases were due to very high values of *τ* measured on the impact before the ‘Crossing’, which prevented detecting a sufficient difference of *τ* between the impact corresponding to the ‘Crossing’ and those corresponding to the ‘Bone’.

55 FP were obtained, which corresponded to impacts belonging to the class ‘Bone’ and classified as belonging to ‘Crossing’.

- For 45/55 FP, the osteotome crossed the bone up to 4 impacts after the FP event. Therefore, these FP events may result from undetectable degradations of the plate, which could be considered as a warning before the osteotome crossed the plate. Out of these 45 FP events, the osteotome crossed the bone 1 (respectively 2, 3 and 4) impacts after the FP for 22 (respectively 12, 7 and 4) impacts.
- For the other 10/55 FP, the osteotome crossed the plate 5 impacts or more after the FP event, which could be explained by the fact that *τ* was high (*τ* > 2.400 ms)

All these 55 FP could be divided into two categories:

1. The actual crossing of the location was detected after this ‘false alarm’. These FP, therefore, allowed to predict the crossing of the osteotome before it occurred. This is the case for 48 FP (see an example in Fig. 4a).
2. No crossing was detected for the corresponding location. These FP were, therefore, a way of detecting the crossing.

For 51/55 FP, the impacts were given in a bone thinner than 0.93 mm and/or a crack was visible on the footage taken with the video camera. This threshold of 0.93 mm corresponded to the intersection of the two regression lines (see Fig. 6 and Table 3) and, therefore, to the minimum thickness which led to an increase of *τ* (see “Relationship Between the Indicator τ and the Thickness” section). As a conclusion, information retrieved from the impact hammer could be used to adapt the force used on the osteotome to the thickness of the pterygoid plate.

This study investigates the use of an instrumented hammer to detect the rupture of the pterygoid plate in a sheep model of pterygomaxillary disjunction. The detection of the osteotome crossing this structure is interesting for maxillofacial procedures such as Le Fort osteotomies. Our approach could prevent unnecessary impacts and therefore avoid complications such as vascular or dental damage. Our results suggest that this technique can be used to detect the osteotome crossing the pterygoid plates and can provide additional information on the thickness of the plate. Future studies should be performed with anatomical subject to be as close as possible to the anatomical and clinical situation.
